# Book Reviews

**Published:** 1820-05

**Authors:** 


### CRITICAL ANALYSIS

### OF

### RECENT PUBLICATIONS, IN THE DIFFERENT BRANCHES OF MEDICINE AND SURGERY.

---

"I would have men know, that, though I reprehend the easie passing over of the causes of things, "by ascribing them to secret and hidden vertues and properties; (for this hath arrested and laid "asleepe all true enquiry and indications;) yet I doe not understand but that, in the practical "part of knowledge much will be left to experience and probation, whereunto indication cannot "so fully reach: and this not only in *specie*, but in *individuo*. Yet it was well said, *Vere scire* "*esse per causas scire*."—BACON.

---

***Pharmacologia; or, the History of Medicinal Substances, with a View to establish the Art of Prescribing and of Composing extemporaneous Formulæ upon fixed and scientific Principles: illustrated by Formulæ, in which the intention of each Element is designated by Key-Letters.*** By JOHN AYRTON PARIS, M.D. F.L.S. Fellow of the Royal College of Physicians of London; Member of the Royal Medical Society of Edinburgh; and late Senior Physician to the Westminster Hospital, &c. &c. Third Edition, very considerably enlarged. 8vo. pp. 429. W. Phillips, 1820.

"Quis Pharmacopœo dabit leges, ignarus ipse agendorum?—Vix profecto dici potest, quantum hæc ignorantia rei, medicæ inferat detrimentum."

GAUBIUS, *Method. concinn. Formul.*

THE author has applied the term *Pharmacologia* to this work, as contradistinctive to that of *Pharmacopæia*, which only denotes the process for preparing medicinal substances; whilst the former comprehends the methods of administering them, and explains the objects and theory of their operation.

The work itself is divided into two distinct parts: one comprehending the principles of the art of combination, illustrated by appropriate examples; the other, the medicinal history and chemical properties and qualities of the bodies which are the subjects of such combination. It is prefaced by a "*historical introduction*," which is the substance of two of the Lectures delivered last year by Dr. Paris before the Royal College of Physicians of London, from the recently-established chair of Materia Medica. This introduction comprises an investigation of the more remarkable causes which have, in different ages, operated in producing the revolutions that characterize the history of medicinal substances.

BACON, when speaking of the persons most subservient to superstition and credulity, puts "sick folks" in the first place; and the truth here indicated will explain, to a great extent, why the history of the use of medicinal substances presents a vaster monument of the extravagancies of human nature, than that of any other subject of their cares, religion only excepted; and, though it has been over the people that the passions abovementioned have exerted their full sway, physicians themselves have not always avoided their influence. Besides this, the means which have given to the other sciences their proper character, and set them in the right road, have failed in their object when applied to this part of human knowledge. *Observation*, *analogy*, and *experiment*, have been those means, exerted in the order in which they are here stated. This the author exemplifies in a very beautiful and forcible manner. OBSERVATION, he says, led NEWTON to discover that the refractive power of transparent substances was, in general, in the ratio of their density; but that, of substances of equal density, those which possessed the refractive power in a higher degree, were inflammable. ANALOGY enabled him to conclude, that, on this account, water even must contain an inflammable principle; and EXPERIMENT enabled CAVENDISH and LAVOISIER to demonstrate the truth of Newton's induction, in their discovery of the chemical composition of this fluid.

But, the application of the above-mentioned precursors of science to the use and effects of medicinal substances is attended with incalculable difficulties, arising out of the variation in their mode of agency, under a multitude of different circumstances of health and disease, constitution, climate, and the fallacious character of the phenomena which appear after their application. Observation and experiment have, indeed, here led to the most extravagant inferences, as Dr. PARIS illustrates by some of those of VOGEL, who professed to assign to substances those powers which had been learned from accumulated experience. He speaks of a *roasted toad*, as a specific for the pains of gout; and asserts that a person may secure himself, for a whole year, from angina, by eating a roasted swallow. Analogy could not be employed with precision until of late years, since the relative perfection of chemistry and botany: the results of its use will engage our attention when we arrive at another part of the work.

The principal causes of the numerous revolutions and vicissitudes presented by the history of this part of medical science, are considered by the author to be superstition, credulity, scepticism; false theory; devotion to authority and established routine; the assigning to art that which was the effect of unassisted nature; ambiguity of nomenclature; the progress of botanical science; the application and misapplication of chemical philosophy, (we should add here, the misapplication of the mathematics;) the influence of climate and seasons on diseases, as well as on the properties and operations of their remedies; the ignorant preparation, or fraudulent adulteration, of medicines; the unseasonable collection of those remedies which are of vegetable origin; the obscurity which has attended the operation of compound medicines:—these causes are successively discussed by Dr. Paris; and he has pourtrayed their effects in an original and very forcible manner. His illustrations of them evince a depth and variety of learning that formerly characterized the writings of the generality of English physicians; but the display of this has lately fallen into disgrace amongst us: that is, since it has been affected by men whose classic reading has not extended beyond the mottos and papers of the *Spectator.* A few examples of the character of that before us, must infallibly soon restore it to its former honourable estimation. The greater part of this portion of the work is not adapted for critical analysis; but we shall adduce a few of the illustrations to which we have just alluded, in order that our exposition of its contents may preserve something like a regular train.

Superstition always exists in a direct ratio to the want of knowledge; and hence the means assumed as remedies for disease, in early ages, in all nations, were principally charms, or expiations to offended spirits, by whom maladies were generally supposed to have been produced. But few practices of this kind now exist amongst the people; yet the reader will be surprised to hear that the character which is placed at the head of our prescriptions, and which is supposed to mean *recipe,* is a relict of the astrological symbol of Jupiter, as may be seen, the author states, in many of the older works on pharmacy, although it is at present so disguised by the addition of the downstroke, which converts it into the letter ℞, that, were it not for its *cloven foot,* we might be led to question the fact of its superstitious origin. The use of superstitious remedies has, however, not been long abrogated by English physicians; for Sir Theodore Mayerne, the *Doctor Caius* of Shakspeare, and who was the most eminent physician of his day, recommends the use of a mummy made of the lungs of a man who had died a violent death, a balsam of bats, and other remedies equally absurd. Our greatest philosophers of the same age were not more free from the influence of this passion; for Boyle discourses on the means by which amulets may cure diseases, by attraction of the morbid particles which have produced them; and he recommends the thigh-bone of an executed criminal as a very powerful remedy. The use of such means was not then merely acceded to, as they are sometimes in the present day by the most judicious physicians, because of their moral influence, but from a firm belief in their supposed physical virtues. Such practices have not, however, always been devoid of beneficial agency, even in a physical manner; for, under the use of Sir Kenelm Digby's *sympathetic powder,* the wound was bound up in a simple way, and *let alone for seven days,*—the surest means for favouring its union by the first intention. The missionaries in

India have also found the utility of superstitious influence in the propagation of cow-pox inoculation.

Credulity, Dr. Paris defines, "an unbounded belief in what is possible, although destitute of proof, and perhaps of probability; whilst superstition is a belief in what is wholly repugnant to the laws of the physical and moral world." Hence the former passion maintains its influence in civilized nations after that of the latter has ceased; and the confidence the one produces in inert substances, is often as ill-founded as that effected by the other, as exemplified by the reputation acquired by a long list of such matters, from the elixir and alkahest of Paracelsus and Van Helmont to the tar-water of Bishop Berkeley. The writings of the later Greek and Roman physicians abound with similar instances; whilst the practices in the temple of Esculapius had been ridiculed as early as the days of Aristophanes and Plautus.

Credulity has been just defined, *belief without reason;* scepticism is its opposite,—*reason without belief;* and is the natural and invariable consequence of credulity: for, it may be generally observed, that men who believe without reason are succeeded by others whom no reasoning can convince: a fact which has occasioned many extraordinary and violent revolutions in the *Materia Medica*, and a knowledge of it will enable us to explain the otherwise-unaccountable rise and fall of many useless as well as important articles.

False theories of the agency of medicines have been the source of a multitude of errors, some of them highly preposterous and ludicrous. This is especially the case with respect to those which have been adopted under the doctrine of *signatures,* which inculcate a belief that *every natural substance which possesses any medicinal virtue, indicates, by an obvious and well-marked external character, the disease for which it is a remedy, or the object for which it should be employed.* Thus, the lungs of a fox must be a specific for asthma, because that animal is remarkable for its strong powers of respiration. *Turmeric* has a brilliant yellow colour, which indicates that it has the power of curing the jaundice: for the same reason, poppies must relieve the diseases of the head; agarius, those of the bladder; cassia fistula, affections of the intestines; aristolochia, the disorders of the uterus; the eagle-stone, worn on the body, prevents abortion; the blood-stone, a bleeding at the nose, &c. &c. Dr. Paris thinks, that John of Gaddesden must have been directed by some remote analogy of this kind, when he ordered the son of Edward the First, who was dangerously ill with the small-pox, to be wrapped in scarlet cloth.

The opposition the progress of medicine has received from devotion to authority and established routine, is too copious a theme to be here dwelt on; we may most of us find sufficient examples of it in our own conduct. The author's illustrations of this subject are pointed and well chosen.

LINNÆUS asserts, in his *Philosophia Botanica*, that the virtues of plants may be generally known from their colour, taste, and smell; and it was once also stated, that the whole of the plants of any certain family possessed similar medicinal analogies: hence the axiom, *Quæ genere conveniunt, virtute conveniunt;* neither of which propositions are strictly true, though they have both tended, in a remarkable degree, to facilitate the progress of the knowledge of the qualities of the materia medica. In objection to the latter, Dr. Paris remarks, that "the *umbelliferæ* which grow on dry ground are aromatic, whilst the aquatic species are amongst the most deadly poisons." This distinction is not however precisely applicable; for celery thrives best in the same soil as that which is most favourable to hemlock. The remark of Dr. Paris is, however, true to a great extent. Linnæus stated that, amongst all the leguminous or papilionaceous tribe, there is no deleterious plant to be found: which is not true; for we have here cystisus laburnum, the seeds of which are violently emetic; those of the lathyrus sativus; and senna cannot be called an innocent plant. In the subdivision even of a genus, there is often a great diversity in the properties of the species: there are, for examples, solanums, lettuces, and cucumbers, both esculent and poisonous. Those whose fruit is a berry present very remarkable differences in their qualities, as shown in the capsicum, common potatoe, the mandragora, and belladonna. All those of the species whose fruit is a capsule, seem, however, to contain more or less of a poisonous principle; as the stramonium, nicotianum, hyoscyamus, verbascum, &c. Perhaps, indeed, the berries of all the solanums, when the plant is not altered by culture, so as to develop the root in an unnatural manner, are more or less of a poisonous nature. The *ranunculacea* present also many diversities of quality; as in the actæa, podophyllum, aconite, hellebore, anemone, the ranunculus properly, and pæonia. We might extend this list to a considerable length, but we must not deviate far from the course taken by the author. The indications of the esculent or medicinal qualities of plants, by their taste, colour, and smell, are somewhat more precise, but cannot be generally relied on. The following observations of Dr. Paris on the bitter quality of plants, seem to us to be remarkably interesting:

"Bitterness, when not extreme, denotes a tonic quality, which will stimulate the stomach and intestines, and promote the powers of digestion. *Bitter extractive* seems to be as essential to the digestion of herbivorous, as salt is to that of carnivorous, animals. It acts merely as a natural stimulant; for it has been shown by a variety of experiments, that it passes through the body without suffering any diminution in its quantity or change in its nature. No cattle will thrive upon grasses which do not contain a portion of this vegetable principle: this has been most satisfactorily proved by the late researches of Mr. Sinclair, gardener to the Duke of Bedford, which are recorded in that magnificent work, the *Hortus Gramineus Woburnensis.* They shew that, if sheep are fed on *yellow turnips,* which contain little or no bitter extractive, they instinctively seek for, and greedily eat, any provender which may contain it; and that, if they cannot obtain it, they become diseased, and die. We are ourselves conscious of the invigorating effect of slight bitters upon our stomach; and their presence in malt liquors not only tends materially to diminish the noxious effects of such potations, but to render them, when taken in moderation, promoters of digestion."

On treating of the application and misapplication of chemical science, the author commences with some well-pointed satire on those men who have imagined interpretations of the mythological symbols of the moral and physical doctrines of the Greeks, applicable to what we now know of this science. BACON conceived that the union of spirit and matter was allegorized in the fable of Proserpine being seized by Pluto as she was gathering flowers: an allusion, says a great man, (whose name we cannot bear to couple with ridicule,) which is rendered more curiously exact by the late discovery, that pure air (oxygen) is given out by vegetables, and that in this state it is greedily absorbed by inflammable bodies. Chemistry, as a science, must be considered entirely of modern origin, according to our present records of its history. Some of the Greeks had, it is true, astonishing notions on this subject: for examples, the theories of HERACLITUS, that water was a compound body, and one of its elements inflammable; on the influence of the sun on the water of the earth; the effects of the evaporation of this fluid, and the origin of clouds and rain from it; the origin of aërial meteors, &c. from gases exhaled from the earth; and many others equally true. But, from its being considered disgraceful for men of intellectual superiority to engage in any mechanical labour, they had no instruments fit for aiding their interrogatories of nature in her more minute and concealed operations; and hence all their knowledge of chemistry as a science consisted in suppositions.

We gave ourselves, a short time since,* a sketch of the history of the application of chemistry to medicine, and pointed out the chief absurdities and false analogies which had thence arisen. Our observations, however, principally related to the application of it to physiology; whilst Dr. Paris examines more especially its connexion with pharmacy. This is a very im-

* In the Number of this Journal for February 1819.

portant part of the present work. It is an interesting fact, that STAHL and BOERHAAVE, two of the best chemists of their age, stated the value of the application of this science to physiology and therapeutics at a very low rate; but they neither of them appreciated it in a precise manner. Dr. Paris appears to state it thus: Chemistry should give laws to pharmacy; but therapeutics should be wholly free from its dominion, excepting only what relates to the decomposition of poisonous or acrid substances present in the intestinal canal. "The mere chemist," he says, "can have no pretension to the art of composing or discriminating *remedies*; whenever he arraigns the scientific propriety of our prescriptions, in direct contradiction to the deductions of true medical experience;—whenever he forsakes his laboratory for the bedside, he forfeits all his claims to our respect, and his title to our confidence." Chemistry is so seductive a science, that most of its real votaries become enthusiasts, and those physicians who have been well versed in it, have generally endeavoured to place it far above its rank amongst the subjects of medicine. There have never been wanting physicians who have expressed sentiments similar to those of Dr. Paris; but the *ultra*-chemists have put in the argument, and it is a forcible one, that they have opposed the utility of a subject, of the principles of which they were ignorant. This cannot be employed on the present occasion; as the author's knowledge of chemistry is deep and comprehensive, and he has himself made several original acquisitions in it; a thing not to be done without at least a moderate degree of love for the science. The opposition of the two parties just designated has led to a sort of careless indecision, in the generality of medical practitioners, respecting the important question under consideration; but, we hope the remarks of Dr. Paris will engage their serious attention, and determine their judgment.

The obscurity which has attended the operation of compound medicines, is another thing which has much retarded the progress of the art of medicine; and, though ORIBASIUS, ALEXANDER of Tralles, VALLISNIERI, and some other of the earlier physicians, had made some interesting observations on this subject, but little light was thrown on it before it engaged the particular attention of FORDYCE, in 1799. Chemistry, and especially the analysis of vegetable substances, had however then made too little progress to enable that great physician to elucidate it in a remarkable manner. As this is a subject of great importance to medical practitioners, they will find the dissertation on it in the work before us highly valuable and interesting to them. We cannot too much praise the views Dr. Paris has taken of it, nor respect too highly the depth of judgment and acute discrimination he evinces in the arrangement of the numerous facts on which his principles are founded.

The objects to be attained by mixing and combining medicinal substances, Dr. Paris states to be:

1°. *To promote the action of the basis, or principal medicine,* which is to be effected, (*a*,) by combining it with substances which are of the *same nature,* that is, which are *individually* capable of producing the same effects, but with less energy than when in combination with each other. Fordyce first demonstrated the existence of the law, that *a combination of similar remedies will produce a more certain, speedy, and considerable effect, than an equivalent dose of any single one:* thus, *emetics* are more efficient when composed of ipecacuanha united with tartarized antimony or sulphate of zinc, than when they consist simply of any one of such substances in an equivalent dose. *Cathartics* not only acquire a very great increase of power by combination with each other, but they are at the same time rendered less irritating in their operation. The *extractum colocynthidis compositum* affords a very good specimen of a compound purgative mass, which is much more active and managable, and less liable to irritate, than any one of its components separately taken. The author furnishes numerous illustrations of the same law, in the several other classes of medicines. The other mean of effecting the first object is, (*b*,) by combining the basis with substances of a *different nature,* and which do not exert any chemical influence upon it, but are found by experience to be capable of rendering the stomach, or system, or any particular organ, more susceptible of its action. As examples, we state the following: Squill is by no means disposed to act upon the urinary organs when exhibited singly, but calomel, and some other mercurial preparations, when in conjunction with it, appear to direct its influence to the kidneys, and to render these organs more susceptible of its operation. Tartar emetic quickens the operation of saline cathartics; opium increases the sudorific powers of antimony; and the purgative operation of jalap is promoted by ipecacuanha. The influence of bark and steel seems to be promoted by the previous use of purgatives. Most of these facts have been remarked by all observant practitioners: we have selected them only as good examples of the influence of the law under consideration. Dr. Paris adduces some others that are original.

The *second* object is, *To correct the operation of the basis, by obviating any unpleasant effects it might be likely to occasion, and which would prevent its intended action, and defeat the objects of its exhibition.* Many important remedies are apt to disagree with the stomach and bowels, and produce vomiting and catharsis, when their influence is principally required on some remote part of the system, and which is thus prevented. The griping and nauseating tendency of some medicines receives correction by the addition of aromatics; the drastic operation of colocynth may be mitigated by trituration with camphor; the griping from senna and resinous purgatives is prevented by the addition of tartrate of potash, or other alkaline salts, by which their solubilities are increased. The foregoing principles are both well exemplified in the *decoctum aloes compositum.* Some substances are deprived of their acrimonious qualities by trituration with mucilage, milk, &c. There is sometimes a *chemical* condition of the stomach, which may interfere with the mild operation of a medicine, and may therefore require consideration: this is particularly exemplified in the action of those antimonial preparations which are liable to become emetic and drastic by the presence of an acid. The vinous infusion of colchicum appears to act more violently when acid is present in the stomach: small doses of magnesia ought therefore to precede, and accompany, its exhibition.

The *third* object is, *To obtain the joint operation of two or more medicines which have different powers, and which are required to obviate different symptoms, or to answer different indications.* To attain this object, correct theoretical views, with much nicety of practical tact, are essentially necessary: if these are wanting, the grossest errors must be committed whenever it is attempted; and, unfortunately, a multitude of practitioners, who hardly ever see a precise indication in any case, commonly resort to the most monstrous and complex combinations, in their intention to attack the several symptoms. We cannot give any thing like an adequate idea of the very important and judicious remarks of the author on this subject in a concise abstract.

The *fourth* object is, *To obtain a new and active remedy not afforded by any single substance.* This is to be effected, (*a*,) by combining medicines which excite different actions in the stomach and system, in consequence of which *new* or modified results are produced; (*b*,) by combining substances which have the property of acting chemically upon each other; the result of which is the formation of *new compounds*, or the decomposition of the original constituents, and the development of their more *active elements.* It may be safely asserted, the author says, that the arm of medicine has derived more power from a few chemical combinations, than from all the numerous simple bodies presented to us by nature, or from the various compounds which art has formed by their simple intermixture; and it is to the crucible and the alembic that we must look for the future improvement and extension of our remedies. The other mean is, (*c*,) by combining substances between which no other chemical change is induced than a diminution or increase in the *solubilities* of the principles which are the repositories of their medicinal virtues.

The *fifth* object is, *To afford a convenient, agreeable, and efficacious, form.* The general and conclusive reflections on the foregoing principles involve a multitude of highly useful remarks on extemporaneous formulæ, and evince the experienced clinical as well as theoretical physician. We can do but little more than adduce some precepts of a general character. We must in the first instance state, that, unless the medical practitioner can satisfactorily trace the operation of each element in his prescription to the accomplishment of one or more of the objects which have just been enumerated, *simplicity* should be regarded by him as the greatest desideratum. Without the knowledge here alluded to, he may lay it down as a maxim, that, in proportion as he complicates a medicine, he does but multiply the chances of its failure. Where the intention of a medicinal compound is obscure, its operation will be uncertain or imbecile.

The chemical and pharmaceutical errors which may be committed in the composition of extemporaneous formulæ, are referable to the following sources: 1°. Substances are added together which are incapable of mixing, or of forming compounds of uniform and suitable consistence. 2°. Substances are added together which mutually decompose each other, whence their original virtues are changed or destroyed. 3°. The methods directed for the preparation of the ingredients are either inadequate to the accomplishment of the object, or they change and destroy the efficacy of the substances.

All the foregoing principles and precepts are illustrated by numerous examples, in a collection of formulæ; and, by an ingenious application of certain key-letters, used as symbols of those principles and precepts, the several prescriptions preserve the ordinary concise form, and yet are in a manner commented on.

The second part of the work is not adapted for analysis. It comprises very accurate descriptions of all the articles of the materia medica; their general physical and chemical properties and qualities; the substances incompatible with them in pharmaceutical combination; their medicinal use; forms of exhibition; adulterations; and the antidotes of such as are poisonous. When we say that this part of the work will be read with considerable interest, even after the perusal of the Dispensatories of Dr. Duncan and Mr. Todd Thomson, we think, we confer on it no small degree of praise. It is full of useful, and often original, remarks on the therapeutical application of the substances of which it gives the history, that will be collected with eagerness by the clinical practitioner; and it makes us regret exceedingly that *pharmacology* has here confined the author's express views. It is, indeed, impossible to peruse this work, without an earnest wish constantly arising, that he had extended them to the *materia medica* as a science. The opinions entertained on this subject are so various or confused, that it is hardly possible to say what are those most generally prevalent. Some, like Brown, consider the essential qualities of the substances as stimulant or sedative to the vital properties, the bases on which all our knowledge in this respect should be raised; as is the case with many English physicians, as well as with the sectaries of the new Italian doctrine, exposed in the Proëmium to the present volume of this Journal. Others think that these should be found in their immediate effects; others, in their more remote effects, evinced in producing expectoration, catharsis, diaphoresis, &c. But the most active disputes are on the question, Whether the *physiological* or the *therapeutical* effects should form the principles of a classification of the materia medica, and the basis on which our use of them should be raised. As the former are constant and identical, except in some rare peculiarities of constitution, and the latter various and uncertain, it would seem that no hesitation should be made respecting the choice of the method that should be adopted. Some one of these views must be better than the rest for the direction of therapeutics; and as, we think, theory is the only basis for a good system of practice, it is much to be desired that some physician of talents and acquirements appropriate for the task would devote his attention to the subject. It is true, that several men of superior intellects, a few years since, laboured assiduously in this field; but the more precise knowledge we now possess in physiology, as well as of the agency of powerful medicinal substances on the human body, and of their chemical constitution, furnish perhaps the means for effecting what they had attempted without success. The French possess some good treatises on this subject, but none of them are precisely what is desired. They are either faulty in theory, or deficient in illustrations drawn from clinical observations. It seems probable that a work might now be produced, that would settle, in general, our conflicting opinions, and constitute a precise and solid basis for future contributions to this part of medical science. Such a work is wanted, to follow the *Pharmacologia*, as well for the instruction of students as for a guide to practitioners; but, to produce one that would be worthy to accompany it, a sort of talents and extent of acquirements are necessary, that are but rarely found in physicians who have leisure and inclination to permit them to pursue so arduous an undertaking.

Dr. Paris, we should state, has treated of the agency of medicinal substances on the animal economy; but his observations on this subject are, generally speaking, only of individual application: they bear a relation to a systematic treatise on the Materia Medica, somewhat similar to that which each of his dissertations on the several articles used as medicines does to his general principles of pharmacy as a science.

---

*Cases of Tic Douloureux, successfully treated.* By BENJAMIN HUTCHINSON, Member of the Royal College of Surgeons of London, &c. 8vo. pp. 71. Longman and Co. 1820.

> Quicquid in arte mea, possum promittere curæ

> Quod fieri FERRO, liquidove potest electro.

THE original discovery of a remedy bearing anything like the character of a specific, is an event that is but rarely recorded at the present day, now that medicine has been unremittingly cultivated by enlightened men for twenty successive ages. The acquisition of such a remedy for any form of disease, however slight, could not be regarded with indifference; for a malady so distressing, and often invincible by all the means by which it had been formerly combatted, as tic douloureux, it must be contemplated with a degree of gratification almost equal to that with which HIPPOCRATES viewed the skeleton of a human body. The existence of an absolute specific for any disease, is like the point without space in geometry, to the experienced medical practitioner of sound judgment, and the author of this work is far from wishing to create such an idea of the remedy he proposes for the affection just designated: his pretensions are never incongruous to the modesty of the title-page of his literary essay; and yet it appears, from his observations, that the remedy has invariably produced considerable relief, if not a perfect cure, of the disease. Such an acquisition does not come by inspiration;—and as, in these times, a surgeon does not, like JAPIS, find ministering divinities bring him *salubris ambrosiæ succos, et odoriferam panaceam*, to heal a gun-shot wound; nor are we taught a new mode of extracting the cataract by goats, in their way of operating on their own eyes with a pointed thorn; nor can a man lie down and dream of a new remedy, even though he had experienced the incantations of a Mesmer;—we are necessarily led, on an occasion like this, to enquire what analogy led to such a discovery. The curative efficacy of arsenic in many cases of tic douloureux, and the failure of it in several instances, only because the poisonous qualities of the mineral prevented the use of it being pursued with sufficient energy, seem to have induced Mr. HUTCHINSON to enquire into the influence of another mineral, which produced effects similar in many respects to those of arsenic, but devoid of its deleterious qualities. This he found in iron: and his expectation of benefit from this substance was increased by the good he had derived from its use, when administered in large doses, in many diseases arising from debility, and from derangement of the functions of the digestive organs. Analogy has not often led to such happy results; and the very worthy physician to whom they are dedicated, and who had himself devoted much of his attention to this disease, must receive the tribute with no small degree of pleasure. The Atlantic Ocean is too wide, to permit Flattery to attempt to send her voice over it to its most distant shores.

Mr. Hutchinson gives a concise account of the history of tic douloureux, and of the measures that had previously been resorted to for its cure, as preliminaries to his original observations. The most efficacious of those measures in the generality of cases, the section of the affected nerves, has produced only temporary relief in the greater proportion of cases, although as efficacious in the first instance as it was in the case of Professor Richerand, related in the present Number of this Journal. This practice has recently been frequently adopted on the continent; but it serves only to show more forcibly the existing despair of the efficacy of the materia medica. Six cases are adduced by the author, to show the efficacy of iron in this disease, under different circumstances of constitution of the patients and the periods of its duration; and he states, that his case-book furnishes him with many others in which its use was equally successful. The preparation he prefers is the carbonate of this mineral, in doses of two scruples, or even a drachm, repeated two or three times a-day; and he remarks, in a corollary way, that it is only necessary to employ it to this extent to remove several other affections, in which it has been supposed to be devoid of beneficial agency. We shall give an abstract of the first case here related, as a specimen of its peculiar powers in the cure of tic douloureux; and, as it is taken from the account the patient herself, an intelligent lady, gives of her malady, the history of it will be particularly satisfactory.

The disease first appeared in the patient's twenty-seventh year: it was seated in the second branch of the fifth pair of nerves, on the right side. The attacks came on suddenly, numerous times in the course of the day, and as suddenly disappeared. It was not attributable to any apparent cause: the health was in other respects good, excepting only the necessary debility arising from the extreme pain, and its preventing the patient taking due sustenance and rest. It continued for twenty years, with but very little respite or intermission. The remedies usually resorted to had been employed during this period without success. At the end of this time she began to take the carbonate of iron, in the quantity of one drachm twice a-day. The relief was immediate. For three years afterwards the attacks occurred only after long intervals, and in but a very slight degree, during the whole of which time the remedy was resorted to for a few days every month; and, for a year previously to the date of the patient's last letter, not a single paroxysm had been experienced.

We shall transcribe the author's history of the treatment of another case, which is given in addition to the patient's own account of her malady, as it will demonstrate the equal efficacy of the remedy in a patient of a different constitution of body from that of the former patient, and in a more acute form of the disease. The subject was a woman about twenty-two years of age, " of a plethoric habit, and of a tense muscular fibre.

" The disease was ushered in by symptoms of active inflammation in the adjacent parts, great pulsation of the arteries of the affected nerves, and considerable febrile irritation. Local and general bleeding, and the usual antiphlogistic modes of treatment, after some considerable length of time, subdued the violence of the inflammatory excitement, without however mitigating in any material degree the excess of her suffering.

" The portio dura of the seventh pair of nerves, spreading its branches to most parts of the face, and communicating with several of those of the fifth pair, distributed to the lower jaw, the mastoid process, and the ear, being here affected, conspired to afflict the patient. She first experienced those peculiar and inexplicable sensations which usually precede a paroxysm: the pains would then assume their most acute and lancinating character, darting along the course of the affected nerves. The periods of their duration varied considerably. The pain did not always confine itself to the seat of the disease, but, as I have before observed, it darted with the velocity of lightning to the neighbouring parts, in various directions.

" Having subdued the highly inflamed state of the parts, I began my assault on this inveterate enemy to the peace of the patient, by the ferri carbonas, in doses of half-a-drachm twice a-day; in which she persevered for the space of three weeks without any considerable amendment of her sufferings. She then became rather weary of taking the medicine. I next tried the efficacy of calomel, and the extract conii, in large doses of the latter ingredient. In this plan I persevered for a fortnight, without producing the slightest impression on the disease. The exhibition of the arsenical solution in tolerably bold doses, was equally unavailing: the sulphate of zinc, the nitrate of silver, the extract of henbane in large doses, shared the same unfortunate results. In this state of depression, I prevailed upon the patient to have recourse to the ferri carbonas, and with a determination to try its power in its fullest dose. I began by giving four scruples twice a-day. On the fifth day after its commencement, a very perceptible change in the disease was evident; the paroxysms were less frequent, and somewhat less severe. A regular perseverance in similar doses during the ensuing month rewarded the patient with a gradual extinction of her malady; and I am happy to add, that she has never experienced the slightest return of it."

The other cases are selected as specimens of the successful use of the remedy in other forms of the disease; and, though the results are apparently inexplicable by theory, the facts they present show its peculiar powers and general efficacy.

---

*A Practical Treatise on Tropical Dysentery, more particularly as it occurs in the East Indies; illustrated by Cases and Appearances on Dissection. To which is added, a Practical Treatise on Scorbutic Dysentery: with some Facts and Observations relative to Scurvy.* By R. W. Bampfield, Esq. Surgeon; Author of an Essay on Hemeralopia, or Night-blindness; and formerly Surgeon of the Belliqueux and Warrior, his Majesty's Ships of the Line, serving in the East and West Indies. pp. 352, and a copious Index. Burgess and Hill, London.

At a period like the present, when dysentery is committing such havoc and devastation among our troops and settlers in India, it becomes extremely gratifying to have to lay before the public our opinion of a work of no inconsiderable value and magnitude, on the subject of that disease; particularly as the author, who has engaged our attention before, has had such ample opportunities of acquiring practical experience in the treatment of the disorder, by a residence of several years in India as surgeon to a line-of-battle ship, with a crew of five hundred Europeans on-board.

The work consists of two parts: dysentery as it occurs within the tropics, and scorbutic dysentery. First, then, as to tropical dysentery. Mr. Bampfield has divided this part of his book into chapters and sections; and he commences by describing the species and varieties which he has observed in tropical dysentery, with great clearness and precision: "and in this enumeration," says our author, "many varieties are omitted which have been described by Dr. Cullen and other nosologists; but the omission has been confined to those which do not require a distinct treatment, or which have not occurred in tropical practice." Thus, he divides them into two species, *dysenteria acuta* and *dysenteria chronica*: to the former he gives three varieties, and to the latter five. Although we believe Mr. Bampfield to be the first writer who has noticed the latter at all as a distinct species, we are convinced, from the accurate manner in which he has described it, that he is fully warranted in so distinguishing it; but it must be here recollected, that Dr. Cullen and his followers, who have omitted to notice this species under a distinct head, had probably never seen this disease within the tropics.

The second chapter is divided into eight sections, and treats of the symptoms that affect the tongue and fauces, the stomach, intestines, liver, urinary and genital organs; of the fever accompanying dysentery; derangement of the heart and vascular system; and, lastly, of the affections of the nervous system. In each of these sections the symptoms are described with great care and minuteness; and, although there are some faults in the work, which we hope to see remedied in another edition, such as referring the reader to cases which he has not given, and the stating others too minutely, of no very considerable degree of interest, yet we consider this work, upon the whole, to be possessed of great merit; as the author appears to be a man of considerable talent for observation, and he has given us ample proofs of his great research: for he seems to have consulted every author who had previously written on dysentery, ancient as well as modern.

On the subject of the tongue and fauces, there is nothing new or very interesting; but, in the next section, namely, on the irritability of the stomach, and sympathies of this organ with the intestines, there are some sensible observations. Frequent nausea and vomiting, he states, are not to be removed until the intestines are emptied by the evacuation of loose fæces, and a free determination of the bile downwards. Attending this state of irritability of the stomach, it is stated, in this part of the work and elsewhere, that there is intense thirst, "which naturally inclines the patient to drink copiously," but the practitioner is guarded against indulging his patient in copious libations; for, if it be to be *retained*, the end in view can only be effected by exhibiting some bland fluid in very small quantities at a time, such as a table-spoonful of barley or rice water, &c. at distinct intervals: and this caution we consider of so much consequence, that we beg to call the particular attention of the reader to it, both in this disease, and in all others where much irritability of this organ exists.

This irritable state of the stomach in dysentery, Mr. B. says,

"Sometimes originates in sympathy with a diseased portion of intestine, or an abdominal viscus. The cause of this irritability cannot always be ascertained; but it has been observed to accompany those cases that have terminated in abscess of some of the abdominal viscera or of their coats. Inflammation of the stomach, or of the little omentum, or of the peritoneum situated over the region of the stomach, will necessarily excite a constant fixed pain about the precordia, and frequent vomiting, and has been sometimes detected as the cause of both. Frequent and unappeasable vomiting and nausea generally succeed the formation of internal abscess.

"In very severe and inflammatory cases, there often exists such an active state of sympathetic irritability through the whole canal, that any warm fluid or stimulant taken into the stomach, will propagate a peristaltic motion through the whole intestinal canal, and bring on torminæ and tenesmus. A direct sympathy also appears to exist, in dysentery, between the stomach and lower intestines; for it often happens that, if the stomach be stimulated by wine, warm food or drink, or aromatic or cordial medicines, an inclination to evacuate, or actual evacuations from the lower intestines, very quickly follow."

With regard to symptoms affecting the intestines, our author goes over nearly the same ground as other writers. It appears, however, that he has only once or twice observed fæces to pass per anum in the form of scybala: on the contrary, when fæces have been passed, either by the action of medicine or otherwise, "they were," he says, "what are commonly denominated loose excrements." This is a peculiarity in tropical dysentery, probably arising from the intestinal secretions being considerably increased in consequence of climate; the secretions in such temperatures being more thin and acrid than in the climate of Europe: and hence scybala are more easily broken down and amalgamated with the more fluid matter, in their passage along the intestinal canal to the rectum.

Sir James Macgregor makes the same remark with regard to the absence of scybala; and we deem this a circumstance worthy of notice to the tyro in tropical practice.

The existence of what Mr. Bampfield calls the inflammatory variety of the disease, is ascertained, he says, by acute and constant pain fixed to some determined spot, whether of the epigastric, hypogastric, umbilical, or pelvic, regions, accompanied by the other constitutional symptoms of local inflammatory action. Our author is particularly desirous to impress upon the reader, what we have ourselves so uniformly urged throughout our critical labours, namely, the great advantage to be derived in practice from a constant and minute examination into the nature and quantity of the evacuations.

The section on the deranged functions, &c. of the liver in dysentery, we think, too short and incomplete: *brevis esse laboro obscurus fio*. Much more might have been said, particularly on the subject of the bile secreted during the existence of dysentery, whether in the severe or mild variety of the disease; and it would be very desirable, if this fluid could be subjected to a chemical analysis, under the circumstances above stated; although we are fully aware of the difficulties attending such a process, particularly on board ships.

"Inflammatory fever (says our author) accompanies some cases of dysentery. In northern, and also in high southern, latitudes, the 'much-increased heat' of synocha is generally attended with a dry skin; in the tropical latitudes, during the hot season, the skin is often moist, and in a state of perspiration: the 'increased heat' of the body must then be inferred and determined from [by] the patient's sensations, and the sense of heat communicated to the medical attendant's hand, when it is applied to a portion of the surface of the body excluded from the contact of external air. In the cold season, the fever is attended with heat and dryness of skin, the same as in Europe.

"It may with truth and justness be observed, that, when inflammatory fever is present, it is almost always accompanied with the other symptoms of inflammation characteristic of the order of phlegmasiæ, &c."

The morbid appearances on dissection form a chapter; but, as there is little in it that has not been noticed by other observers, we shall pass on to the part which treats of predisposition, and the causes of tropical dysentery; being the most interesting and valuable part of the work under review, if we except the latter part, which embraces the important subject of treatment. We may observe, however, *en passant*, that we see no reason why the remote and proximate causes of dysentery might not have formed different sections under the head of predisposition, instead of their being thrown into two distinct chapters.

Mr. Bampfield's theory on the subject of predisposition, and his ingenious reasonings on the sympathies existing between the skin and the whole of the chylopoietic viscera, are so clear and forcible, that, as we are led onwards, we feel as strong a conviction in favour of our author's view of the subject as can possibly be felt upon any theoretical point, supported by the soundest arguments and logical discussions.

In this section Mr. Bampfield withdraws the veil a little that has too long covered the egotistical assumptions and unscientific notions of Dr. James Johnson, which are contained in his work on Tropical Climates; and here we were led to refer to the work itself, to see what Dr. Johnson has said on the points to which our author alludes; and we find that, in no less than three or four different places, namely, at pages 13, 15, and 165, Dr. Johnson claims as a *discovery* a particular sympathy, which he states to exist between the skin and the liver, and which he has not inaptly termed cutaneo-hepatic. But, who before ever heard of a theory, however ingenious, being designated as a discovery? Mr. Bampfield quotes Sir Gilbert Blane on the Diseases of Seamen, as having noticed the sympathies now under consideration, upwards of thirty years ago; but we can refer our author and Dr. Johnson to much older authority than that mentioned, and, without going through a whole catalogue of dignified names in the profession, we shall only mention Darwin, Harvey, and Glisson; the latter having been very appropriately termed the father of the doctrine of sympathies; he was physician to Queen Elizabeth, and celebrated for his anatomical discoveries and sound physiological doctrines.*

It appears to us, that Mr. Bampfield is perfectly correct in stating that this sympathy, or consent of parts, said to exist between the skin and liver, ought to be called the *reverse* sympathy, after Darwin, and not a synchronous association of action, as stated by Dr. Johnson; for, in the former, analogy bears one out in their reasoning; and to argue that, when the perspiration is profuse, the secretion of the bile is at the same time "regularly, and to appearance equally, increased,"† is to argue that nature's laws are diametrically different in one gland from that of another. We know that, as it regards the secretion in the kidneys, whenever perspiration is profuse, micturition is proportionably diminished, and *vice versa*. Here we bring forward striking analogy in support of our opinions; and, when Dr. Johnson does the same, we shall then consider how far he may be entitled to style himself a discoverer of something bordering upon a fact. But we have strayed from our way.

The whole of Mr. Bampfield's observations and reasonings on the subject of predisposition to, and the causes of, tropical dysentery, are so highly interesting and valuable to all descriptions of readers, that we earnestly recommend this part of the book to every individual in or out of the profession, whose interest or inclination may lead them to visit the Tropics. To select one passage in preference to another, would be an injustice equally to the reader and to the author.

*On the treatment of dysentery.*—In this important part of the work Mr. Bampfield has arranged, in systematic order, the various remedies had recourse to in this disease, beginning with that which in his practice has been the most powerful, viz. blood-letting. He commences the chapter by stating, that

"The indications of cure in acute dysentery, are, to reduce or subdue inflammatory action by general and topical bleeding, and those remedies that diminish the frequency of the pulse; and to restore the lost balance of the circulation, by increasing other secretions and excretions, but more especially of those organs, glands, mucous membranes, and capillary vessels, whose actions and functions are associated with the diseased intestines: of the healthy intestine, by cathartics; of the skin, by sudorifics and recumbent posture; of the bladder, [*quere*, kidneys?] by diuretics and diluents; of the liver, by calomel; and of the salivary glands, if necessary, by mercury, the action of which is peculiarly powerful and efficacious in reducing minor degrees

* See his work *De Ventriculo et Intestinis;* and his other work, entitled *Anatomia Hepatis.*

† See Dr. Johnson's book, second edition, p. 13.

of inflammation. Besides these indications, constipation must be speedily and effectually removed, and palliative remedies be employed for particular symptoms. The utility and propriety of some of these remedies must be distinctly considered."

This short sentence seems to us to embrace every medicinal curative means necessary to be had recourse to in dysentery, and requires judgment only in the timely application of them. The arrangement is as follows: bleeding, cathartics, diaphoretics, mercurial preparations, regimen, palliative remedies for particular symptoms, &c. With respect to the first, we have of late years so uniformly seen the necessity of this remedy, both by the dissections related by authors, and the favourable result of the practice by those few who have pursued it, that we cannot recommend it in too strong terms to the attentive consideration and adoption of our numerous readers; and we particularly refer such of them as may be sceptical on this subject, to Dr. SOMERS's valuable Treatise on Dysentery, as it occurred among our troops in the Peninsula, and to Mr. BALLINGALL's work, entitled "Practical Observations on the East-India Dysentery," published about the time the work now before us was in the press: in both which essays will be found the strongest evidence as to the propriety and necessity of early and decisive blood-letting in this disease, according to the rules of practice laid down by Mr. Bampfield.

Cathartics are recommended to be constantly had recourse to by our author, excepting in such cases of dysentery as have been immediately preceded by copious diarrhœa; in which case, "some respite from their use may be granted. They are not only necessary to remove the constipation attendant upon dysentery," but are highly useful, by exciting an increased secretion from the mucous membrane of the healthy intestine. Draughts are recommended by Mr. B. in preference to any other formula, when the stomach will retain them; such as a solution of the magnesiæ sulphas in aqua menthæ, or infusum sennæ, sharpened in their action by a minute quantity of tartarized antimony; or, he says, the ol. ricini may be given with advantage. We confess ourselves to be in favour of the latter, as being more lubricating, and less likely to add to the inflammatory action already existing in some part of the intestines: but, where the stomach is irritable, we are of opinion, that from six to nine grains of calomel, with or without a grain of scammony, exhibited every four hours in some tenacious vehicle, would be more likely to be retained; for, even if vomiting were to occur afterwards, it would be less likely to be rejected than any other medicine with which we are acquainted, both on account of its weight and adherence to the coats of the stomach; but we should not recommend more than thirty or thirty-five grains of the submuriate to be given in this way, and which having failed in producing feculent motions, we should then proceed to administer other purgatives noticed in the work: and here we deem it proper to mention, before we take our leave of this part of the subject, that we have found castor-oil retained in very many instances, when every other purgative medicine has been rejected by the stomach. "To demonstrate," says Mr. B. "the obstinacy of the constipation, and the necessity of persevering activity, I shall observe, that many cases have occurred in my practice which required six, eight, or ten, ounces of sulphas magnesiæ vel sodæ, with twenty or thirty grains of hydrarg. submurias, to produce fecal evacuations." Warm mucilaginous and demulcent drinks are recommended during the period that purgatives are being administered, both to assist them in their action and defend the diseased intestine from acrid stimuli.

Stimulating cathartic enemata our author very justly reprobates; but mucilaginous, oleaginous, or starch, enemas, are highly useful, by sheathing the intestines, acting as internal fomentations, and soothing pain. After very full and copious evacuations of fæces, anodyne enemas are extremely necessary and beneficial, particularly when tenesmus and torminæ are severe. Of late years, syringes have been commonly used for the purpose of throwing up enemas; but, from long and extensive experience, we are of opinion that, in dysentery or enteritis, when injections have become necessary, the common bag and pipe is by far the preferable instrument, because much less air is thrown up into the intestine by it than by the syringe; and, in these diseases, air pent up in the alimentary canal we all know to be a very distressing symptom to the patient. One word more on the subject of cathartics, and we have done. As a complete cure in dysentery can only be effected by a full and free discharge of feculent matter from the intestines, and this kept up throughout the whole period of the existence of the disorder, we cannot insist too much on the frequent repetition of such medicines as will effectually promote the end in view; and, in very obstinate cases of constipation, we have not unfrequently assisted the operation of aperient medicines by friction over the abdomen with the naked hand or a piece of fine flannel, and also by the use of the warm bath.

With regard to regimen, it is very properly remarked, that animal food should almost always be abstained from, and farinaceous substances those only which the patient should be indulged in, such as sago, arrow-root, tapioca, rice, &c.

"The utility of diaphoretics," says Mr. B. "appears to be diminished in proportion to the previous habit of profuse perspiration established by the climate, which is agreeable to the laws of the animal economy. Indeed, in the hot season, mere confinement to bed, with warm diluents and covering, will maintain profuse perspiration, and should be practised." He is however far from thinking, with Dr. HARTZ, that perspiration alone will cure dysentery: nay, he has noticed instances, he says, in which profuse perspiration has been injurious. Mr. B. does not object to, or condemn, the practice, but merely cautions "the practitioner not to expect too much benefit from it; for I am of opinion," he says, "a gentle moisture should be always maintained on the surface of the body, that we may be assured the disease is not protracted, nor increased, by the operation of the remote cause of checked perspiration; and that every advantage may be derived from the sympathy established between the skin and intestines."

During the cold season of the year, in India and China, diaphoretic medicines become more necessary, the degree of temperature being then below what will procure a natural moisture on the surface. We must then have recourse to artificial means; and with this view Mr. Bampfield recommends ipecacuanha, pulvis antimonialis, or antimonium tartarizatum, combined with calomel or other remedies. These, he states, may be assisted by warm diluents, warm bath, pedeluvium, a recumbent posture of the body, and warm covering.

In this part of the work Mr. B. takes occasion strongly to recommend the application of blisters to the abdomen; observing, that they will be found fully as useful in dysentery as in other complaints of an inflammatory character.

We are next led on to the use of mercurial preparations; and, in the discussion on this subject, Mr. Bampfield evinces great good sense, and inculcates sound practical doctrines to the tropical practitioner. He states, that very few cases died where ptyalism had been excited; but that, where much inflammatory action existed, ptyalism could not be effectually induced. "Hence," he says, "the necessity of previous bleeding and purging in the inflammatory variety; for, when high inflammation is subdued by them, no obstacle will oppose the induction of mercurial irritation, and its beneficial consequences: besides, mercurial irritation often excites general increased action of the heart and arteries, and would consequently be injurious," &c. He goes on to observe, however, that all minor or moderate degrees of visceral inflammation may be subdued by the exhibition of this mineral. A review is next taken of the various preparations to be employed; and the hydrargyri submurias stands the first in the list, which he recommends to be combined with other purgative medicines, or with minute doses of the pulvis ipecacuanhæ. The unguent hydrargyri fortius is used, he says, with a view to produce ptyalism, and the blue pill as a substitute for calomel, when, from some peculiarity of constitution, the latter preparation disagrees with the patient.

These observations are followed by some directions concerning regimen, and remedies to be employed for particular symptoms, and such as are considered of a palliative nature; when we arrive at what may be deemed the second division of this useful work, *chronic dysentery.* Our author treats of the several varieties of chronic dysentery at great length, and with his usual discrimination. We must however here take our leave of the work, having already devoted more than the usual number of pages to its consideration.

The last part of Mr. Bampfield's book, entitled *Observations on Scorbutic Dysentery*, &c. we must likewise leave unnoticed; strongly recommending a perusal of it to all navy surgeons and surgeons of East-India ships, as containing more interesting matter on this species of dysentery, as well as on scurvy in general, than is to be met with in any other book with which we are acquainted.

To sum up our opinion of Mr. Bampfield's work in a few words: as a whole, we consider it to be our imperative duty to say, that, as a practical treatise, it should hold the first rank in every medical man's library who may be called upon to exercise his professional duties within the Tropics, and who has that regard for the safety of his patients, and his own reputation, which every honourable mind ought to possess.

---

***A Treatise on the Nature of Scrofula, in which an Attempt is made to account for the Origin of that Disease on new Principles; illustrated by various Facts and Observations, explanatory of a Method for its complete Eradication: together with an Appendix, containing several interesting Cases.*** **By William Farr, Surgeon, Member of the Royal College of Surgeons, London; and late Surgeon to the Hospital in the Island of Anholt. 8vo. pp. 76. Wright, 1820.**

The author of this book is, it appears, unacquainted with either the practice of English surgeons in general in the disease of which he here treats, the best opinions entertained by them respecting its nature, or the spirit which directs them in the cultivation of their art. The Preface alone is sufficient to show the propriety of two of the above remarks, and the rest of the book will prove that of the other. He says, "Theory, rather than practice, has engrossed the attention of preceding writers;" that he is conscious of the difficulties he has to combat, and the disadvantages he labours under, "as well from the reserve with which any new practice is received by a considerable part of the profession, as the prejudices entertained by some of its members, prematurely considering any deviation from the beaten track as an unjustifiable innovation on established rules:" and this flattering picture of the mode of prosecuting the medical art amongst us, and of our liberality and zeal for its improvement, concludes with the remark, that he has "been much more solicitous to merit approbation by the elucidation of facts and solidity of reasoning, than for purity of style or elegance of composition."

This assertion, and the statement in the title-page, must lead the reader to expect a new theory of scrofula, as well as an improved mode of treating it: and it is true, that Mr. Farr has produced something apparently intended to be a theory; but, for *new principles* and *solidity of reasoning*, we have sought in vain. After stating that "it must be evident to the merest tyro in physiology, that some important operation in the chylopoiëtic system" is accomplished by the mesenteric glands, he says,

"Reasoning from the fact I have just stated, may it not be inferred, that, in all enlargements of these glands, without the existence of that morbid alteration of structure which would necessarily render them impervious, such alteration of function exists, as, by its action on the passing chyle, so materially changes its properties, as to lay the foundation for struma? This materia morbi, the offspring of such impaired action in these glands, though I do not class it in the number of specific poisons, as far as regards the production of a similar disease by inoculation, may, from its action upon the constitution of the individual in whom it is generated, by inducing that diathesis so favourable to the existence of scrofula, be considered at least an exciting, if not the proximate, cause of the disease.

"By some, hereditary predisposition has been said to consist in a peculiar structure of the whole body, or some particular parts of it, or that it is a something, the *rationale* of which no one can satisfactorily explain; but that it is transmitted from parents to their offspring, and constitutes the *sine qua non* of the malady. I certainly do not consider its agency as bearing that extreme application which some are willing to admit, namely, that it should necessarily follow that children whose parents have been afflicted with the disease must suffer, in consequence of their unfortunate title to it from inheritance: neither am I more disposed to coincide in opinion with those who admit the influence of hereditary disposition, and assert that its effects should be uniform in all children of the same family; but, that it does operate in a modified sense, the following narration will sufficiently demonstrate."

The *narration* shows merely what was already universally known: that some children of a scrofulous father may be affected with the disease, whilst others will escape, either from not having a connate disposition to it, or from having been removed during their infancy to a more salubrious climate than that inhabited by those who had become its victims. The whole of the rest of what relates to the nature of the disease, is made up either of the merest common-place matter, or, in those parts where reasoning is attempted, of such a confused compilation of ideas and mass of false inferences, that criticism lies down the pen before it.

Mr. Farr's mode of treating the disease consists in the use of caustic alkali and mercurial frictions. The alkali has received a very extensive trial by English surgeons; and in one of the London hospitals its use has been pursued, for a series of years, in the generality of cases. According to the observations of almost all practitioners, mercury has been found injurious rather than beneficial in scrofula; and it is very common to see this affection either originally developed, or aggravated, by the use of mercury for other diseases. Mr. Farr relates twelve cases which terminated favourably under the measures above designated; but they are not so accurately related, as to permit us to conclude that they were all cases of scrofula; and, were this proved, they would only show that some cases may do well under measures which are ordinarily injurious. There is nothing original or worthy of particular notice, for its merit, in the more minute parts of Mr. Farr's practice. We recommend Mr. Farr to peruse what has been written on scrofula by our best authors; and, as we believe him to be directed by honourable motives, we feel confident, that, if he follow our advice, and subsequently write on that disease; the egotistical tone he has here assumed, in stating what is vulgarly known; the citations he has made of obsolete notions, instead of those of late years prevalent amongst well-informed men, as specimens of the opinions of his contemporaries; and the display he makes of the merest common-place and most trivial measures, as new and important discoveries; will not again be the traits which characterize his production.

